# Differentiation of bisphenol F diglycidyl ether isomers and their derivatives by HPLC-MS and GC-MS—comment on the published data

**DOI:** 10.1007/s00216-021-03157-2

**Published:** 2021-01-20

**Authors:** Małgorzata Kasperkowiak, Monika Beszterda, Izabela Bańczyk, Rafał Frański

**Affiliations:** 1 Center for Advanced Technology, Adam Mickiewicz University, Uniwersytetu Poznańskiego 10, 61-614 Poznań, Poland; 2 Department of Food Biochemistry and Analysis, Poznań University of Life Sciences, Mazowiecka 48, 60-623 Poznań, Poland; 3 Faculty of Chemistry, Adam Mickiewicz University, Uniwersytetu Poznańskiego 8, 61-614 Poznań, Poland

**Keywords:** Bisphenol F diglycidyl ether, Isomer distribution, Liquid chromatography, Gas chromatography, Mass spectrometry, Fragmentation pattern

## Abstract

Positional isomers of bisphenol F diglycidyl ether (BFDGE) have been analyzed by high-pressure liquid chromatography-mass spectrometry and by gas chromatography-mass spectrometry (HPLC-MS, GC-MS). Positional isomers of BFDGE derivatives (BFDGEx2H_2_O, BFDGExH_2_OxHCl) have been analyzed by HPLC-MS. On the basis of the obtained fragmentation patterns, the elution order of the isomers has been unequivocally determined, in standard solutions and in the sample of liquid obtained after rinsing an empty mackerel fish can with acetonitrile. Under HPLC condition, *para,para* isomers are eluted first, then *ortho,para* isomers’ elution follows, and *ortho,ortho* isomers are eluted last. Under GC condition, the reverse elution order has been obtained. For the first time, two *ortho,para* isomers of BFDGExH_2_OxHCl have been detected and their elution order has been determined. The obtained results are of key importance for determination of the isomer distribution of BFDGE and its derivatives in food samples.

**Supplementary Information:**

The online version contains supplementary material available at 10.1007/s00216-021-03157-2.

## Introduction

The subject area of plastic materials is inseparably connected to issues concerning bisphenol A and its analogues. Unfortunately, much less attention has been given to bisphenol-diglycidyl ethers (BDGEs), which are also used for the production of these materials. Because of their widespread use, the endocrine-disrupting potentials, mutagenicity, and teratogenicity observed in in vitro and in vivo studies, BDGEs were suspected of posing health risks to humans, especially to infants [1, 2]. Bisphenol F diglycidyl ether (BFDGE) is a synthetic industrial compound obtained by the reaction between novolac and epichlorohydrin. It is used mainly for the production of epoxy resins, as an additive to polyesters and a hydrogen chloride binding agent during varnished surface degradation [3, 4]. Epoxy resin blends are commonly used as internal lacquers for direct food contact in food cans and this compound may be washed off the material surface and transferred to food matrix or individual elements of the environment [5].

BFDGE, in contrast to the other bisphenols, almost always exist as a mixture of positional isomers, namely *para,para*-BFDGE, *ortho,para*-BFDGE, and *ortho,ortho*-BFDGE. High-pressure liquid chromatography-mass spectrometry (HPLC-MS) has been successfully used for analysis of BFDGE. Some authors have reported not perfect but acceptable separation of isomers [6–11], while others in order to provide information about the isomer distribution in the analyzed samples have succeeded in perfect separation of isomers [12–17]. However, the identification of the isomers has not been performed. In order to provide information about the isomer distribution, the elution order has to be determined, and in our opinion, it has not been unambiguously performed yet, as discussed further in details.

Gas chromatography-mass spectrometry (GC-MS) has been also used for analysis of BFDGE isomers, although definitely less often than HPLC-MS [18–23]. It is expected that the isomers have substantially different melting and boiling points. Furthermore, the electron ionization (EI) mass spectra of the isomers should be also different; therefore, they should be easily separated and identified by GC-MS. However, we found that their analysis by GC-MS is sometimes disputable, as discussed further in details.

In this work, it has been shown how the BFDGE isomers should be differentiated by HPLC-MS and GC-MS and the elution order of BFDGE isomers under HPLC-MS and GC-MS conditions has been established. The products obtained by hydrolysis of epoxy group, namely BFDGEx2H_2_O and BFDGExH_2_OxHCl, have been included in the HPLC-MS study (GC-MS is not suitable method for the analysis of BFDGE hydrolysis products). We have also analyzed a number of the extracts of canned foods and BFDGE isomers have been identified in the sample of empty mackerel fish can rinsing with acetonitrile.

## Materials and methods

### Chemicals and sample preparations

BFDGE (CAS: 2095-03-6) has been obtained from Sigma-Aldrich. In order to obtain the hydrolysis products, 1 mg of BFDGE was placed in 1 ml of 5% water solution of hydrochloric acid and stirred for 10 min. in ultrasonic bath. Because of the low BFDGE solubility in water, the obtained solution was cloudy. After 48-h storage at room temperature, the solution became almost clear indicating that the reaction with H_2_O/HCl occurred. In these conditions, two hydrolysis products were formed, namely BFDGEx2H_2_O and BFDGExH_2_OxHCl. The third hydrolysis product, namely BFDGEx2HCl, was formed in small amount and has not been included in the study.

A total number of 6 canned foods, such as sea foods in oil, meat products, and newborn and infant formulas, were purchased from local markets in western Poland. The empty cans were filled completely with acetonitrile (Super Purity Solvent, Romil Ltd) and the extraction process was carried out by carefully stirring the solution for 90 min. The extracts were concentrated to a minimum by vacuum evaporation (Rotavapor R-120, Büchi) and made up to a final volume of 3 ml with acetonitrile. Prior to the HPLC/ESI-MS analysis, the sample was further filtered through syringe filters with a pore size of 0.45 μm (Macherey-Nagel GmbH, Germany). The food samples were first homogenized in the flow homogenizer (H 500, Pol-EkoAparatura, Polska). Each homogenized sample (50 g) was extracted twice with 100 ml of hexane (for HPLC, Avantor Performance Materials Poland) for 20 min at gradually increasing speed to 20,000 rpm using laboratory shaker (358S, Elpin plus, Poland). The samples were then filtered over a funnel on cellulosic fluted filters into a round-bottom flask. The hexane was evaporated by vacuum rotary evaporation (Rotavapor R-120, Büchi, Switzerland) to a minimum. The round-bottom flask was rinsed with a total volume of 10 ml of hexane used in aliquots and transferred to the centrifuge vial. After 5 ml of acetonitrile was added, the centrifuge vials were then shaken for 90 min and centrifuged at 2600*g* for 10 min. The acetonitrile phase was then taken up with an automatic pipette after removal of the hexane phase, filtered through syringe filters (the same as above) to vials. The presence of BFDGE was only identified in the sample of empty mackerel fish can rinsing with acetonitrile.

### HPLC-MS method

The HPLC-ESI-MS analyses were performed using a UltiMateTM 3000 UHPLC system (ThermoScientific/Dionex) and Impact HD mass spectrometer (q-tof type instrument equipped with electrospray ion source; Bruker Daltonics). Using an autosampler, the sample solutions were injected onto the Kinetex C18 column (100 × 2.10 mm i.d., 2.6 μm particle size; Phenomenex). The used mobile phases were water with 0.1% of formic acid (solvent A) and acetonitrile with 0.1% of formic acid (solvent B). The flow rate was 0.3 ml/min and the column temperature was maintained at 35 °C. The solutions were analyzed by using linear gradient of CH_3_CN-H_2_O; both solvents contained 0.1% formic acid. The gradient started from 30% CH_3_CN, reaching 65% CH_3_CN after 25 min, and the latter concentration was maintained for 5 min. In the above conditions, abundant [M+NH_4_]^+^ ions were generated. Usually, in order to generate abundant [M+NH_4_]^+^ ions, the mobile phase contains ammonium salt; however, we found that it is not necessary (of course addition of ammonium salt may yield better sensitivity/separation). The mass spectra were recorded in positive ion mode. The instrument was operated under the following optimized settings: end-plate voltage 500 V, capillary voltage 4.2 kV; nebulizer pressure 1.0 bar; dry gas (nitrogen) temperature 200 °C; dry gas flow rate 8 l/min. The spectrometer was previously calibrated with the standard tune mixture. The product ion spectra obtained during HPLC-ESI-MS/MS analysis were obtained at collision energy of 13 eV.

### GC-MS method

GC-EIMS analysis was performed using SCION GC-436 gas chromatography system coupled to a TQ mass spectrometric detector (Bruker Daltonics, Germany; triple quadrupole mass spectrometer). Samples were separated using a BP5MS capillary column coated with 5% phenylpolysilphenylene-siloxane (30 m × 250 μm i.d., 0.25 μm film thickness; SGE Analytical Science). The initial oven temperature was held at 80 °C for 2 min, ramped to 290 °C at a rate of 15 °C min^−1^ and then held for 7 min. Helium was used as a carrier gas at a constant flow rate of 1 ml min^−1^ through the column. The temperatures of the injector, transfer line, and ion source (EI) were set at 210, 250, and 200 °C, respectively. The ionization energy was 70 eV. The mass spectral data was collected in a full scan mode (*m/z* 35–500).

## Results and discussion

### HPLC-MS analysis

It has been proposed by Gallart-Ayala et al. that *p,p*-BFGDE isomer is eluted first, since the fragmentation of ion [M+NH_4_]^+^ of this isomer yielded only two product ions at *m/z* 163 and 107 and the latter product ion was not observed upon the fragmentation of ion [M+NH_4_]^+^ of other isomers [24]. However, this fragmentation of *p,p*-BFGDE isomer has not been confirmed by literature data in which all three isomers have been analyzed, by product ions at *m/z* 163 and 133 (the product ions were formed during the fragmentation of ions [M+NH_4_]^+^ at *m/z* 330) [6, 7, 13, 15]. Yang et al. have proposed that *o,o*-BFDGE isomer is eluted first; however, their claim was not rationalized [15].

It is reasonable that there are two possible elution orders, namely *p,p*-BFDGE, *o,p*-BFDGE, *o,o*-BFDGE, or reverse. It is strongly expected that fragmentation pattern of *p,p*-BFDGE will be different than that of *o,p*-BFDGE and *o,o*-BFDGE. For *o,p*-BFDGE and *o,o*-BFDGE, the so-called *orth*o effect should take place. It is well known that electron ionization mass spectra of 1,2-disubstituted aromatic compounds are substantially different from those of their positional isomers. There are a number of examples that in ESI conditions the *ortho* effect also takes place [25–31].

**Fig. 1:**
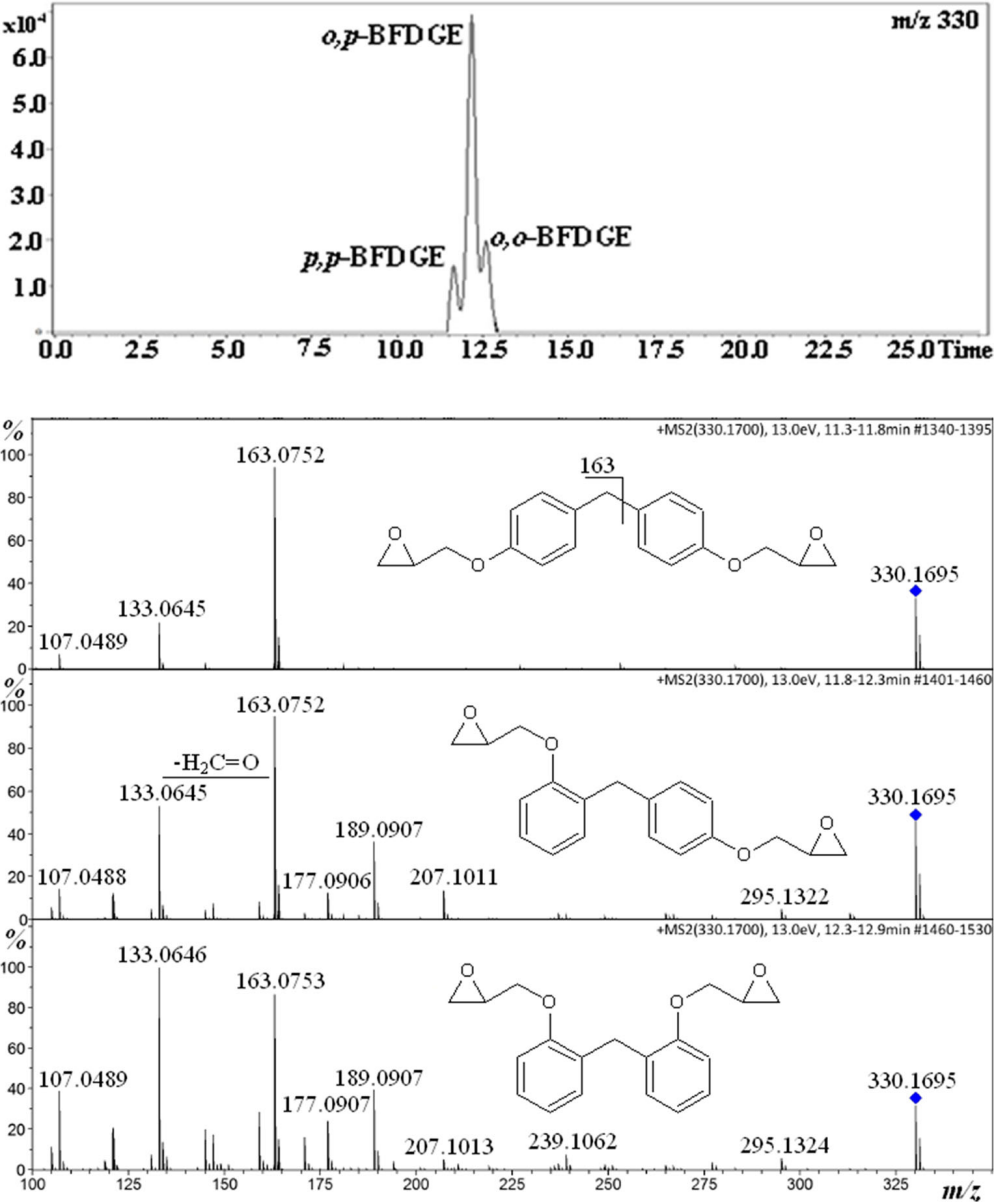
Single ion chromatogram of BFGDE [M+NH_4_]^+^ ion and product ion spectra of the isomers (standard solution, HPLC-MS analysis)

Figure 1 shows the single ion chromatogram of [M+NH_4_]^+^ ion (*m/z* 330) and the product ion spectra of [M+NH_4_]^+^ ions obtained for BFDGE solution. The separation of the isomers is acceptable and allows getting product ion spectrum for each of them.

It is clear that the mass spectrum of the isomer which has been eluted first is substantially different than the mass spectra of other isomers. Therefore, the elution order is *p,p*-BFDGE, *o,p*-BFDGE, *o,o*-BFDGE. In the mass spectra of *o,p*-BFDGE and *o,o*-BFDGE isomers, there are a number of peaks of product ions which are absent in the mass spectrum of *p,p*-BFDGE isomer (the most abundant is that at *m/z* 189; Fig. 1). However, it is rather difficult to rationalize why the product ions are formed as a result of *ortho* effect, but this is out of the scope of this paper, for example, how to rationalize the formation of product ion at *m/z* 295 which is formed as a result of the loss of H_2_O and NH_3_ molecules from [M+NH_4_]^+^ ions of *o,p*-BFDGE and *o,o*-BFDGE isomers. It has to be stressed that the obtained accurate masses of product ions are in full agreement with those reported by Gallart-Ayala et al. [24].

**Fig. 2:**
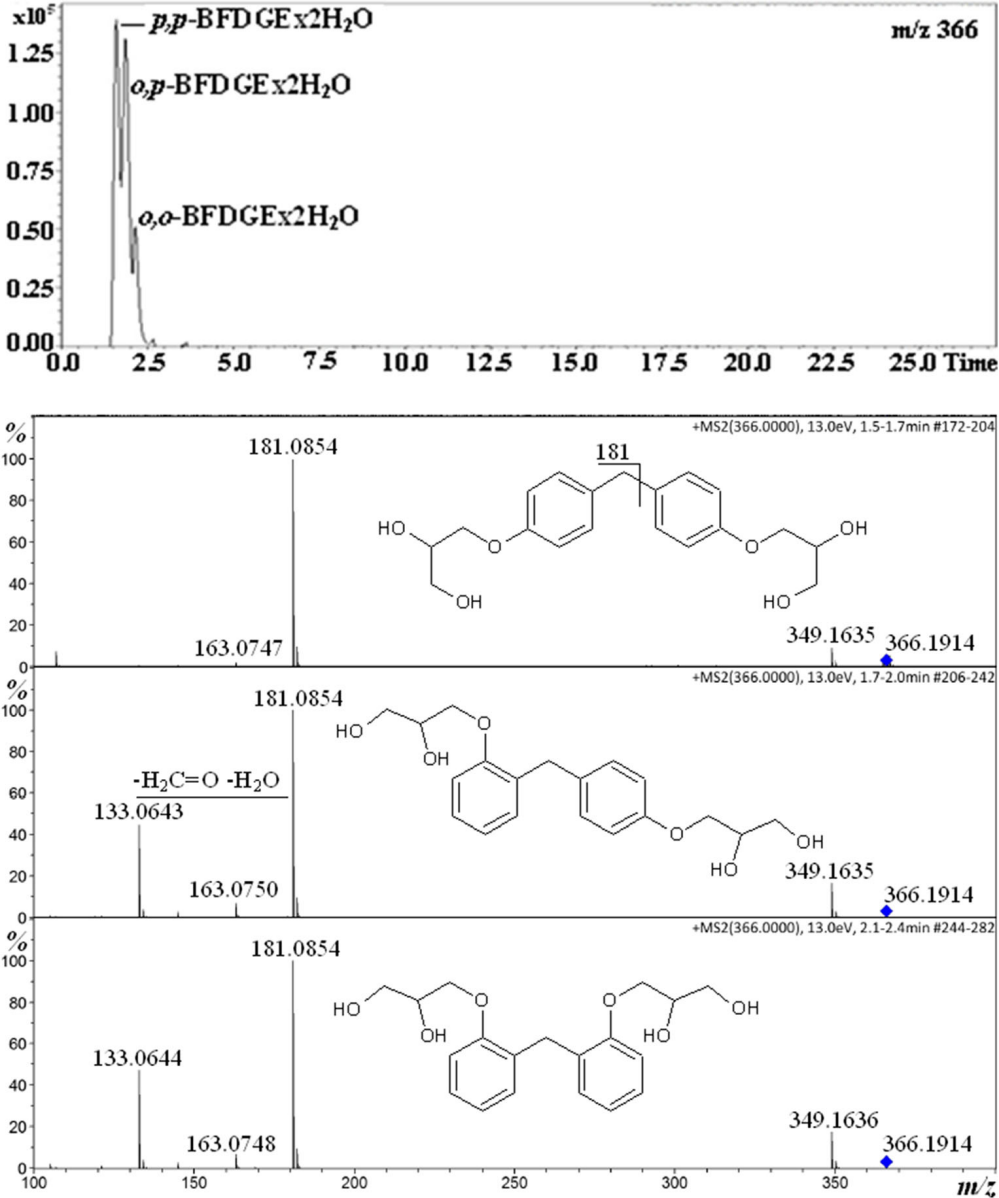
Single ion chromatogram of BFGDEx2H_2_O [M+NH_4_]^+^ ion and product ion spectra of the isomers

Fig. 2 shows the chromatogram and product ion spectra obtained for BFGDEx2H_2_O isomers. Analogically as for BFDGE isomers, the mass spectrum of the isomer which has been eluted first is substantially different than the mass spectra of other isomers, namely there is no product ion at *m/z* 133 for the first eluted isomer. Therefore, the elution order is *p,p*-BFDGEx2H_2_O, *o,p*-BFDGEx2H_2_O, *o,o*-BFDGEx2H_2_O.

**Fig. 3:**
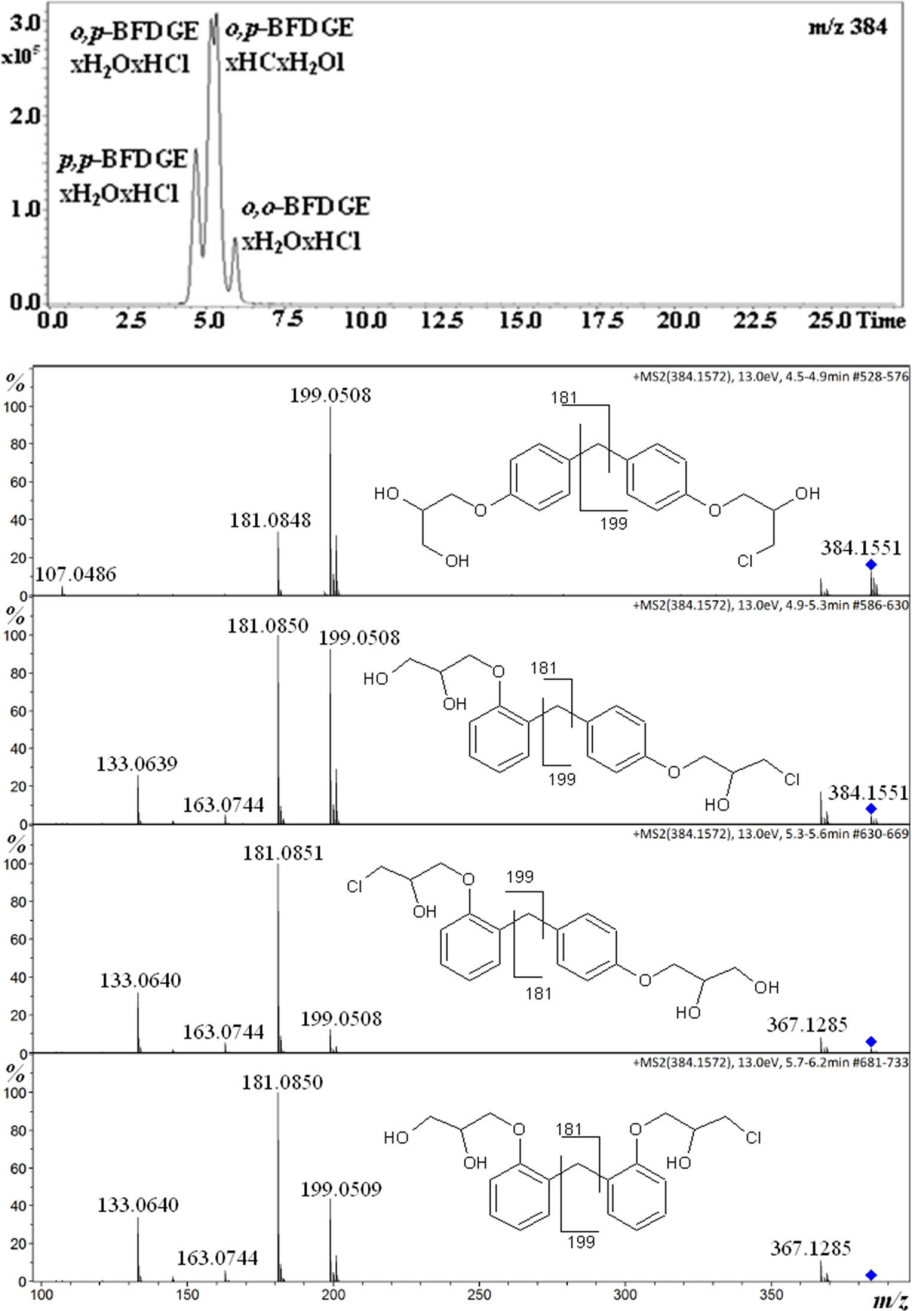
Single ion chromatogram of BFGDExH_2_OxHCl [M+NH_4_]^+^ ion and product ion spectra of the isomers

Figure 3 shows the chromatogram and product ion spectra obtained for BFGDExH_2_OxHCl isomers. We have obtained four isomers, namely *p,p*-BFDGExH_2_OxHCl, *o,p*-BFDGExH_2_OxHCl (Cl containing the moiety at *para* position), *o,p*-BFDGExHClxH_2_O (Cl containing the moiety at *ortho* position), and *o,o*-BFDGExH_2_OxHCl. The first eluted isomer is *p,p*-BFDGExH_2_OxHCl, since only for this isomer there is no product ion at *m/z* 133. Analogically as for BFDGE and BFGDEx2H_2_O isomers, the mass spectrum of the isomer which has been eluted first is substantially different than the mass spectra of other isomers. It is reasonable that the *o,p*-isomers are eluted as the second ones (Fig. 3). Although the separation of *o,p*-isomers is poor, it was possible to obtain mass spectrum for each of them. On the basis of relative abundances of ions at *m/z* 181 and 199, it was possible to deduce that the first eluted isomer is *o,p*-BFDGExH_2_OxHCl (Fig. 3). Namely, for *p,p*-BFDGExH_2_OxHCl *m/z* 199 > *m/z* 181 and for *o,o*-BFDGExH_2_OxHCl *m/z* 199 < *m/z* 181, therefore, it is clear that *o,p*-BFDGExH_2_OxHCl has higher relative abundance of *m/z* 199 than *o,p*-BFDGExHClxH_2_O (Fig. 3).

It has to be added that most of the papers devoted to the analysis of BFDGE and its derivatives is focused on the BFDGE, BFDGEx2H_2_O and BFDGEx2HCl, whereas BFGDExH_2_OxHCl was analyzed only occasionally [9, 16, 32]. It may be due to the lack of respective standards. On the other hand, it is undisputable that during the formation of BFDGEx2H_2_O and BFDGEx2HCl, the BFGDExH_2_OxHCl must be formed as well. Furthermore, to the best of our knowledge, the formation and separation of *o,p*-BFDGExH_2_OxHCl and *o,p*-BFDGExHClxH_2_O isomers have not been reported yet.

**Fig. 4:**
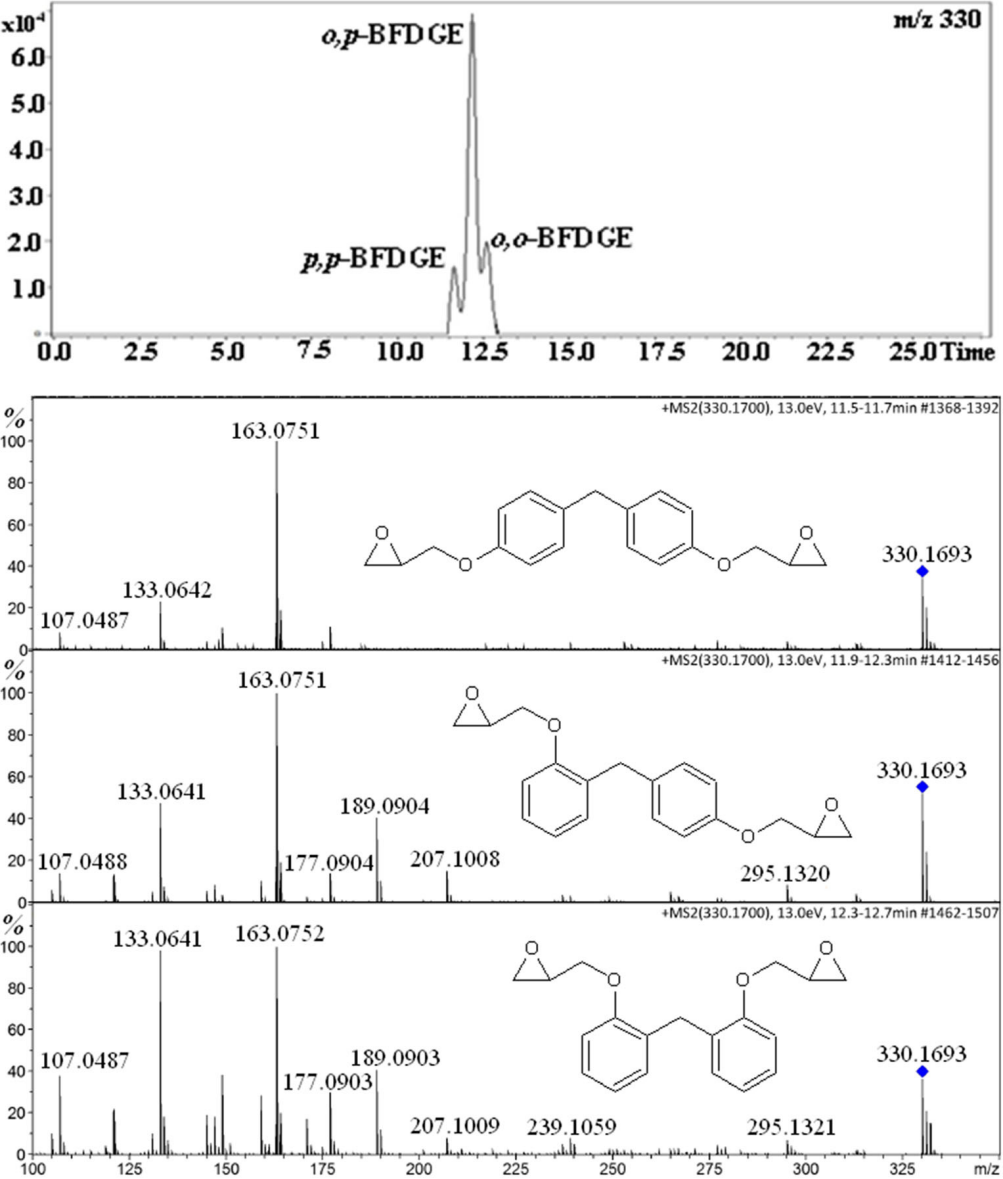
Single ion chromatogram of BFGDE [M+NH_4_]^+^ ion and product ion spectra of the isomers obtained for acetonitrile extract of empty mackerel fish can

We have also detected the BFDGE isomers in acetonitrile extract of empty mackerel fish can; however, the BFDGE derivatives have not been detected. As shown in Fig. 4, the isomer distribution is different than that in the standard solution. Namely the relative content of *o,p*-BFDGE is much higher than those of *o,o*-BFDGE and *p,p*-BFDGE.

In the supplementary material, there are the results of the HPLC/ESI-MS analysis performed under different conditions than those described above and the elution order of BFDGE isomers and derivatives, deduced on the basis of the mass spectra obtained, is the same as that described above.

The chromatograms shown in Figs. 1, 2, and 3 and those in Figure S1 (Supplementary Information) have been obtained for the same solution; however, the relative abundances of the isomers are different. It is known that the relative ion abundances under ESI condition may be affected by the number of factors, e.g., pressure inside the instrument and solution composition (e.g., the concentration of contaminants Na^+^, NH_4_^+^). Therefore, the conclusion can be drawn that the relative responses of the discussed isomers, under ESI condition, are very sensitive to the condition used. Thus, the conclusion concerning the relative isomer distribution should be drawn with caution. In other words, the conclusion should be drawn on the basis of the analysis performed in identical HPLC-MS conditions.

### GC-MS analysis

**Fig. 5:**
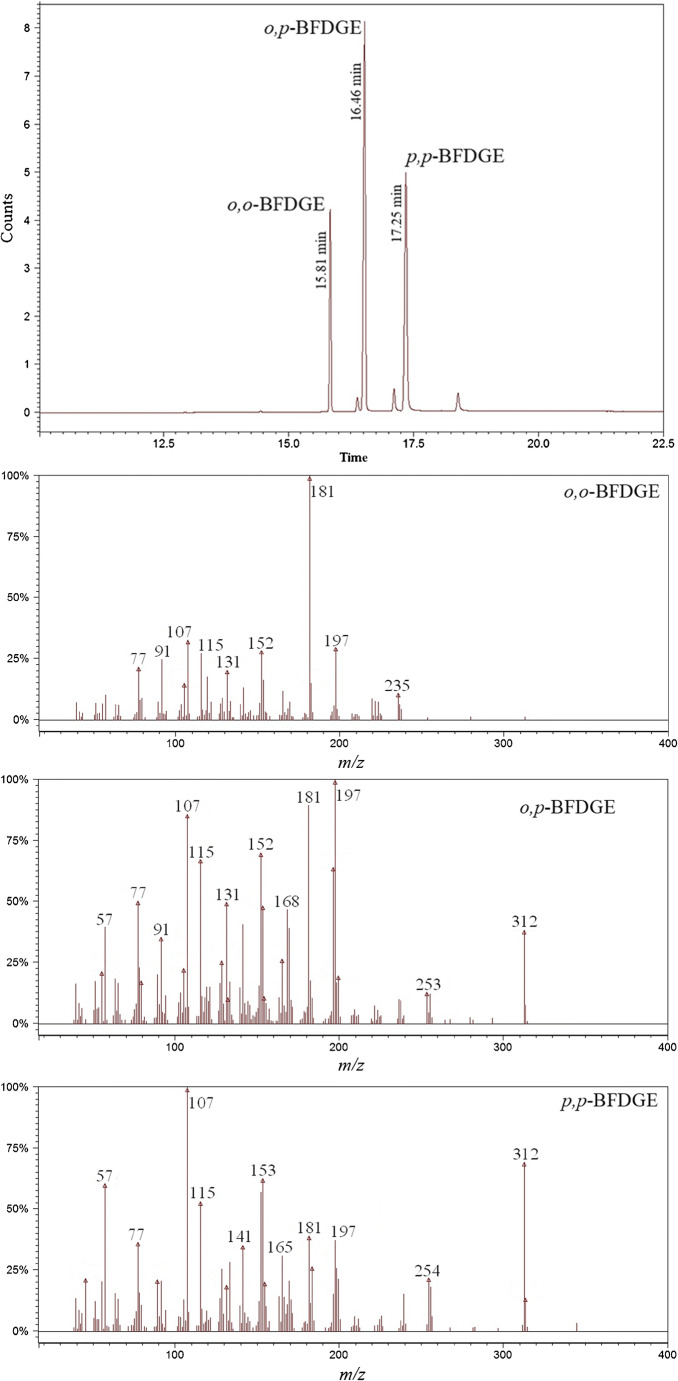
Total ion chromatogram obtained for BFDGE isomers and EI mass spectra obtained (standard solution, GC-MS analysis)

Figure 5 shows the obtained chromatogram for a solution of BFDGE isomers and their EI mass spectra. The isomers are well separated. It is strongly expected that the elution order is *o,o*-BFDGE, *o,p*-BFDGE and *p,p*-BFDGE (the more branched compound, the lower the melting/boiling point, the shorter the retention time) [33]. By comparing the chromatograms shown in Fig. 1 with that shown in Fig. 5. it is clear that we deal with different relative responses of the isomers under HPLC-MS and GC-MS conditions. It is not surprising, since as mentioned earlier, even under different HPLC-MS conditions, the isomers can have different relative responses.

The obtained EI mass spectra of the isomers are substantially different and confirm the elution order (Fig. 5). For the isomers *o,o*-BFDGE and *o,p*-BFDGE, the *ortho* effects are clearly seen. Isomer *o,o*-BFDGE does not yield the molecular ion (*m/z* 312) and the most abundant fragment ion is that at *m/z* 181, indicating its high stability. For the *o,p*-BFDGE isomer, the *ortho* effect is manifested by the formation of the abundant fragment ion at *m/z* 197. Plausible structures of fragment ions at *m/z* 181 and 197 are shown and briefly discussed in supplementary material. For isomer *p,p*-BFDGE, the most abundant fragment ion is that at *m/z* 107. It can be easily calculated that the ion is hydroxy-substituted, well-known, benzylium/tropylium ion (HO-C_6_H_4_-CH_2_^+^).

The results shown in Fig. 5 indicate that the GC-MS identification of BFDGE positional isomers should be trivial (of course provided that the concentration in the analyzed sample is higher than the detection limit of the instrument used). The elution order and EI mass spectra similar to those shown in Fig. 5 have been obtained by Vílchez et al., although the authors have not assigned the isomer structures to the spectra and chromatographic peaks [18]. The small discrepancy, which is worth noting, is that Vílchez et al. have observed the molecular ion for *o,o*-BFDGE (*m/z* 312, not abundant but clearly seen). The EI mass spectra obtained at different instruments should be similar but do not have to be identical. Brede et al. have also indicated the same elution order of BFDGE isomers as that in Fig. 5 [19]. All the isomers were detected by the ions at *m/z* 181, 197, and 312 (molecular ion). Brede et al., besides the BFDGE isomers, have also analyzed BADGE (bisphenol A diglycidyl ether) and it has been identified only by the fragment ion at *m/z* 325 (CH_3_ loss from molecular ion); thus, molecular ion of BADGE has not been detected by Brede et al. [19]. It is a little surprising that Brede et al. have observed the molecular ion of *o,o*-BFDGE, whereas they have not observed the molecular ion of BADGE. We have observed the molecular ion of BADGE, similarly as Vílchez et al., as shown in the supplementary material. In spite of the above-described small discrepancies (one of the reason may be different source temperatures used, 280 °C by Vílchez et al., 300 °C by Brede et al., and 200 °C in our work), our results and those obtained by Vílchez et al. and by Brede et al. clearly indicate the elution order of BFDGE isomers as well as which ions should be used for detection/differentiation of BFDGE isomers. The EI mass spectra obtained at different instruments should be similar but do not have to be identical. In the supplementary material, there are the results of GC-MS analysis of BFDGE isomers performed for standard solution by using an ion trap mass spectrometer. The obtained relative responses of the isomers, by using two different GC-MS instruments (chromatograms shown in Fig. 5 and S6), are not identical, although the differences are definitely smaller than in the case of HPLC-MS instruments (Fig. 1 and S1).

**Fig. 6:**
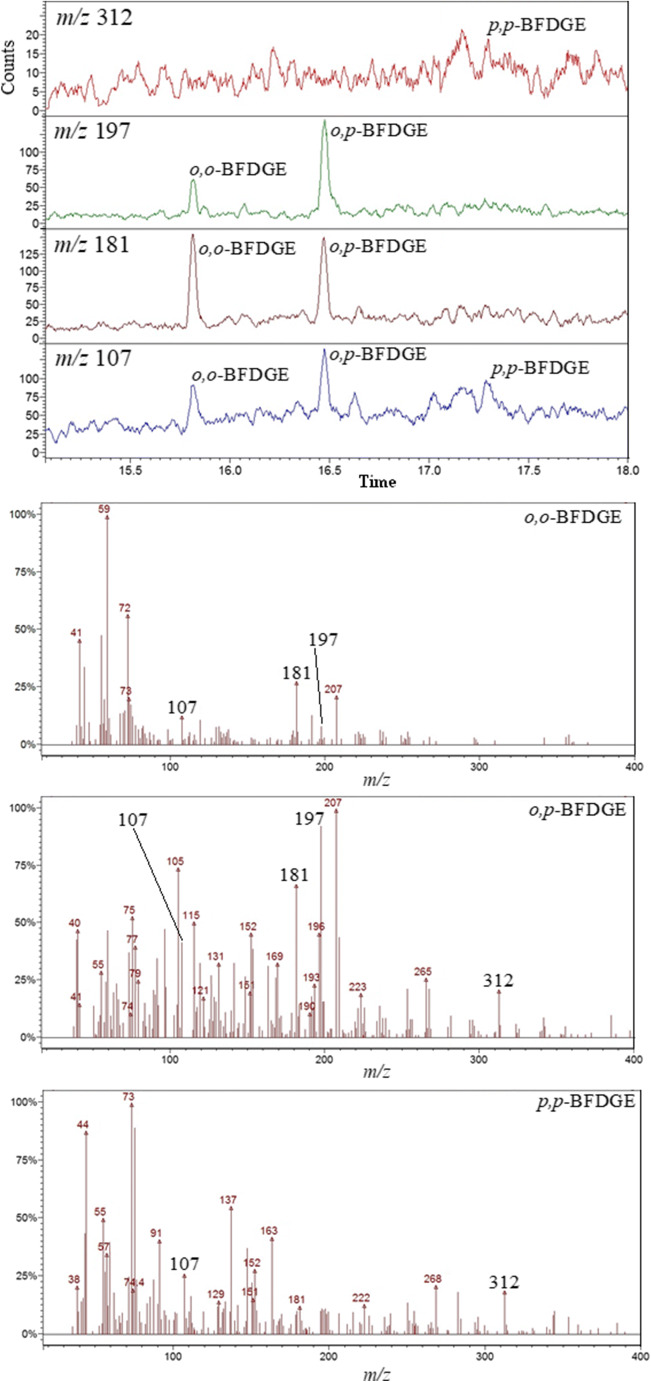
Single ion chromatograms of BFGDE derived ions and EI mass spectra obtained for acetonitrile extract of empty mackerel fish can (GC-MS analysis)

Figure 6 shows single ion chromatogram and mass spectra obtained for acetonitrile extract of empty mackerel fish can.

It is clear that *o,o*-BFDGE and *o,p*-BFDGE have been detected in acetonitrile extract just above of the detection limits (the high background is observed). The relative abundances of *o,o*-BFDGE and *o,p*-BFDGE derived ions (*m/z* 107, 181, 197, 312) confirm the presence of the isomers in acetonitrile extract, by comparison with Fig. 5. The detection of *p,p*-BFDGE in acetonitrile extract is disputable (Fig. 6).

Some of the published literature data concerning GC-MS analysis of BFDGE are a little disputable. Oca et al. have observed abundant molecular ion and 100% relative intensity for fragment ion at *m/z* 197, for the first eluted isomer, thus for *o,o*-BFDGE isomer [20, 21]. The authors have claimed that it is the *p,p*-BFDGE isomer. In our opinion, the peak relative intensities reported by Oca et al. correspond to the *o,p*-BFDGE isomer. Jiao et al. have identified the BFDGE isomers by using the fragment ions which have low abundances in comparison with the other fragment ions, namely *m/z* 168 for *o,o*-BFDGE, *m/z* 141 for *o,p*-BFDGE, and *m/z* 168/198 for *p,p*-BFDGE [22]. Jordákova et al. have analyzed all the BFGDE isomers by molecular ion and by fragment ion at *m/z* 207, which is not characteristic for the BFGDE isomers [23]. It is worth adding that the authors of [20–23] have used the GC columns of polarity similar to that used in our work (e.g., Oca et al. have used HP-5MS); thus, the different elution order than that obtained in our work cannot be expected. It has to be stressed that all the papers commented here (including those by Gallart-Ayala et al., Oca et al., Jiao et al., and Jordákova et al.) are characterized by high scientific level, the procedures described in the papers can find practical application, and our very specific comment does not discredit them in any way.

## Conclusions

It was clearly demonstrated that the BFDGE isomers and their derivatives can be differentiated by HPLC-MS and by GC-MS, while the hydrolysis products of BFDGE isomers can be differentiated by HPLC-MS. Therefore, the distribution of BFDGE isomers and derivatives in food samples can be determined. For each of the analyzed isomers, under HPLC conditions, the elution order was *p,p*-isomer, *o,p*-isomer, *o,o*-isomer. Assuming that it could be possible that under specific HPLC conditions the elution order would be different, the elution order should be easy to determine, as described above (including differentiation between *o,p*-BFDGExH_2_OxHCl and *o,p*-BFDGExHClxH_2_O). Under GC conditions, the elution order was *o,o*-BFDGE, *o,p*-BFDGE, and *p,p*-BFDGE. Under GC condition, any different elution order cannot be expected. Both under ESI and EI conditions, the fragmentation patterns of *p,p*-isomers are substantially different than those of *o,p*- and *p,p*-isomers, for which the characteristic *ortho* effects have been observed.

## Supplementary information

ESM 1 (PDF 831 kb)
