# Supplementary material for: Differentiation of bisphenol F diglycidyl ether isomers and their derivatives by HPLC-MS and GC-MS—comment on the published data
Source: Anal Bioanal Chem. 2021 Jan 20;413(7):1893–903. doi: 10.1007/s00216-021-03157-2 (PMC7925483; doi:10.1007/s00216-021-03157-2)
Supplement: Supplementary file 1 — (PDF 831 kb) [file 216_2021_3157_MOESM1_ESM.pdf]

Supplementary Information

Differentiation of bisphenol F diglycidyl ether isomers and their derivatives by HPLC-MS and GC-MS—comment on the published data

HPLC-MS analysis

The HPLC-ESI-MS analyses were performed using a Waters model 2690 HPLC pump (Milford, MA, USA), a Waters/Micromass ZQ2000 mass spectrometer (single quadrupole type instrument equipped with electrospray ion source, Z-spray, Manchester, UK). The software used was MassLynx V3.5 (Manchester, UK). Using an autosampler, the sample solutions were injected onto the C18 Atlantis T3 column (3 µm, 100 mm x 3 mm i.d.; Waters, Warsaw, Poland). The sample concentration was 0.1 mg/mL and injection volume was 1 µL. The solutions were analyzed by using linear gradient of CH_3CN-H_2O or CH_3OH-H_2O with a flow rate of 0.4 mL/min. The gradient started from 0 % CH_3CN (or CH_3OH) – 95 % H_2O with 5 % of a 10 % solution of formic acid in water, reaching 95 % CH_3CN (or CH_3OH) after 30 min, and the latter concentration was maintained for 10 min. Thus the full time of HPLC/ESI-MS analysis was 40 min, however, for clarity, the chromatograms are shown for a smaller time range.

The ESI mass spectra were recorded in the m/z range 100-1000. The ESI source potentials were: capillary 3 kV, lens 0.5 kV, extractor 4 V and cone voltage 20–50 V. Cone voltage has the most profound effect on the mass spectra obtained. Increase in this parameter leads to the so-called "in-source" fragmentation/dissociation but a too low cone voltage may cause a decrease in sensitivity. The chromatograms and mass spectra shown further are those obtained at 30-40 V. The source temperature was 120 °C and the desolvation temperature was 300 °C. Nitrogen was used as the nebulizing and desolvation gas at flow rates of 100 and 300 L/h, respectively.

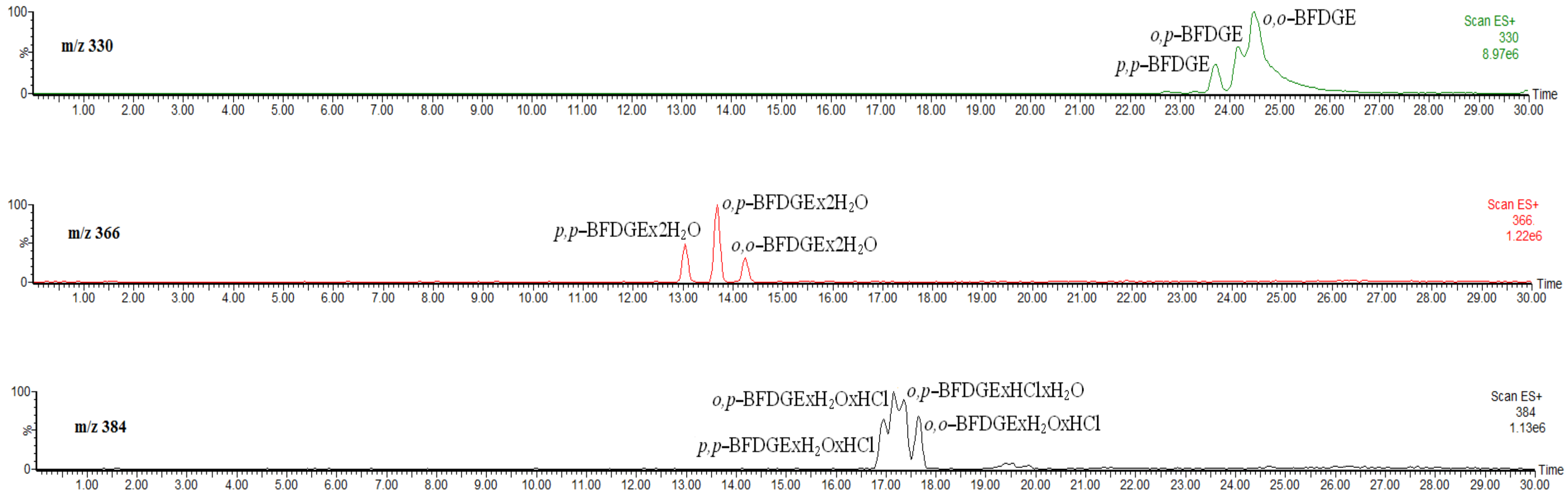

Fig. S1 Single ion chromatogram of $[M+NH_4]^+$ ions (CH_3OH-H_2O gradient for BFDGE, CH_3CN-H_2O gradient for derivatives)

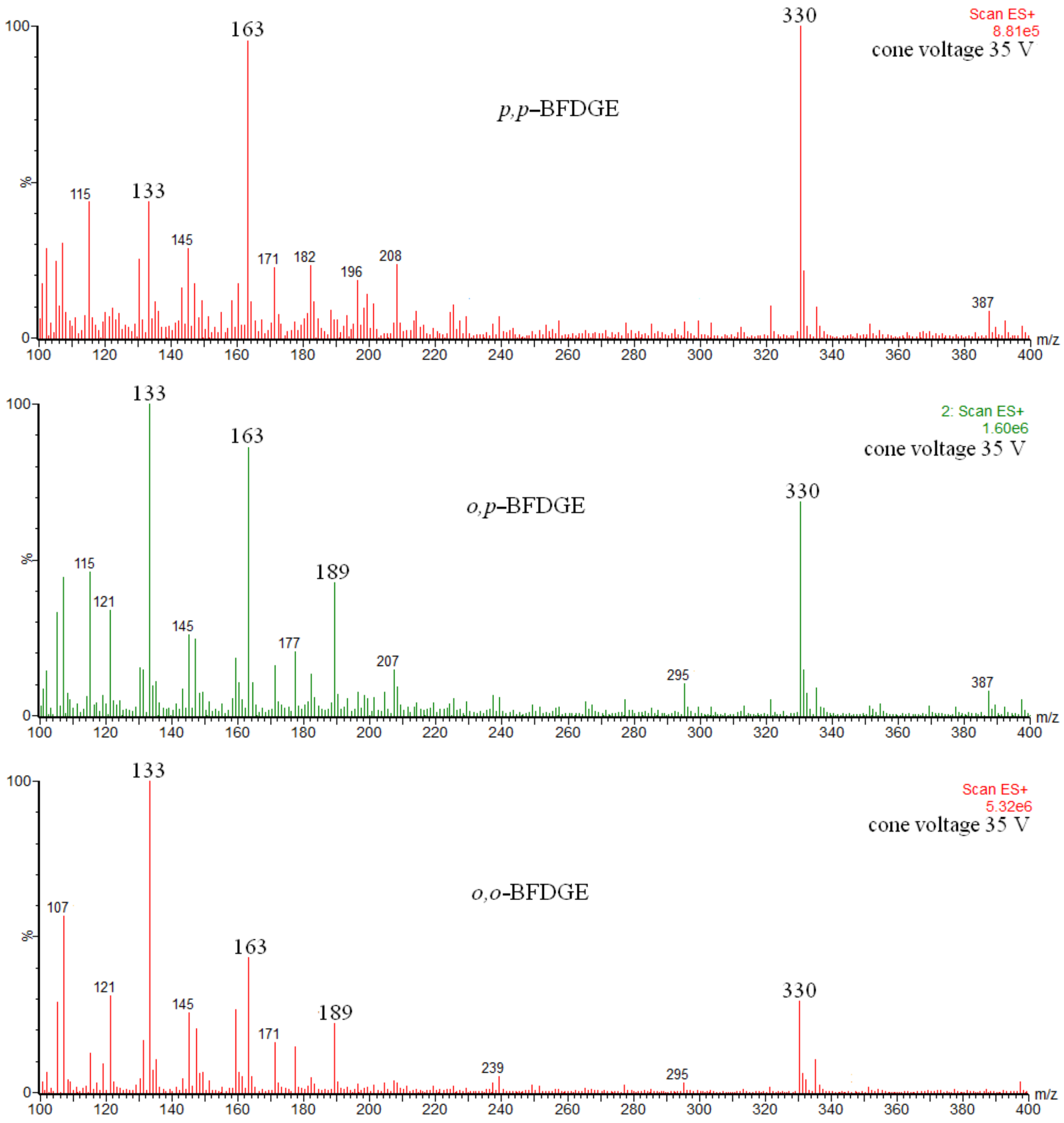

Fig. S2 ESI mass spectra of BFDGE isomers ($[M+NH_4]^+$ *m/z* 330)

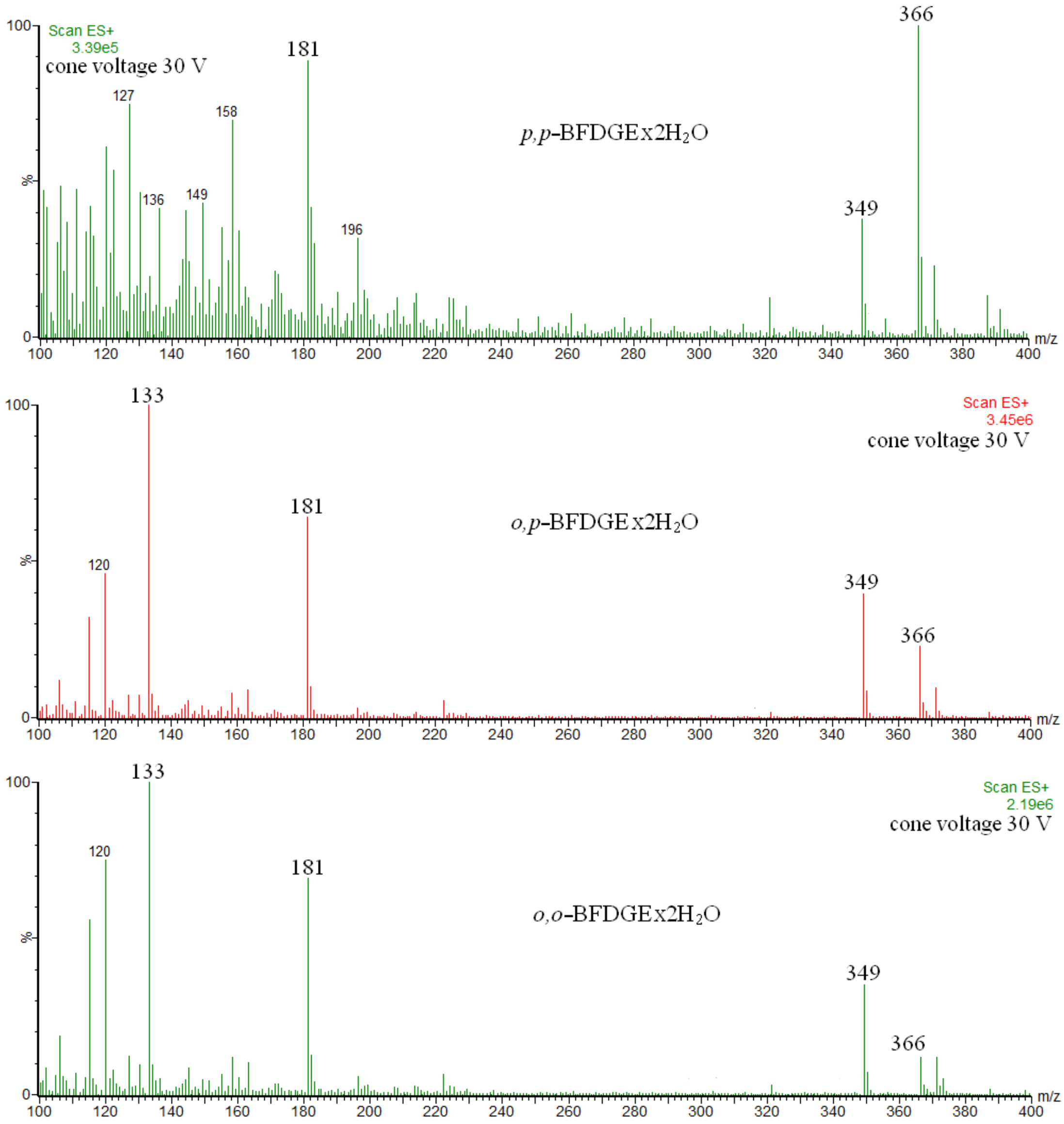

Fig. S3 ESI mass spectra BFGDEx2H_2O isomers ($[M+NH_4]^+$ *m/z* 366)

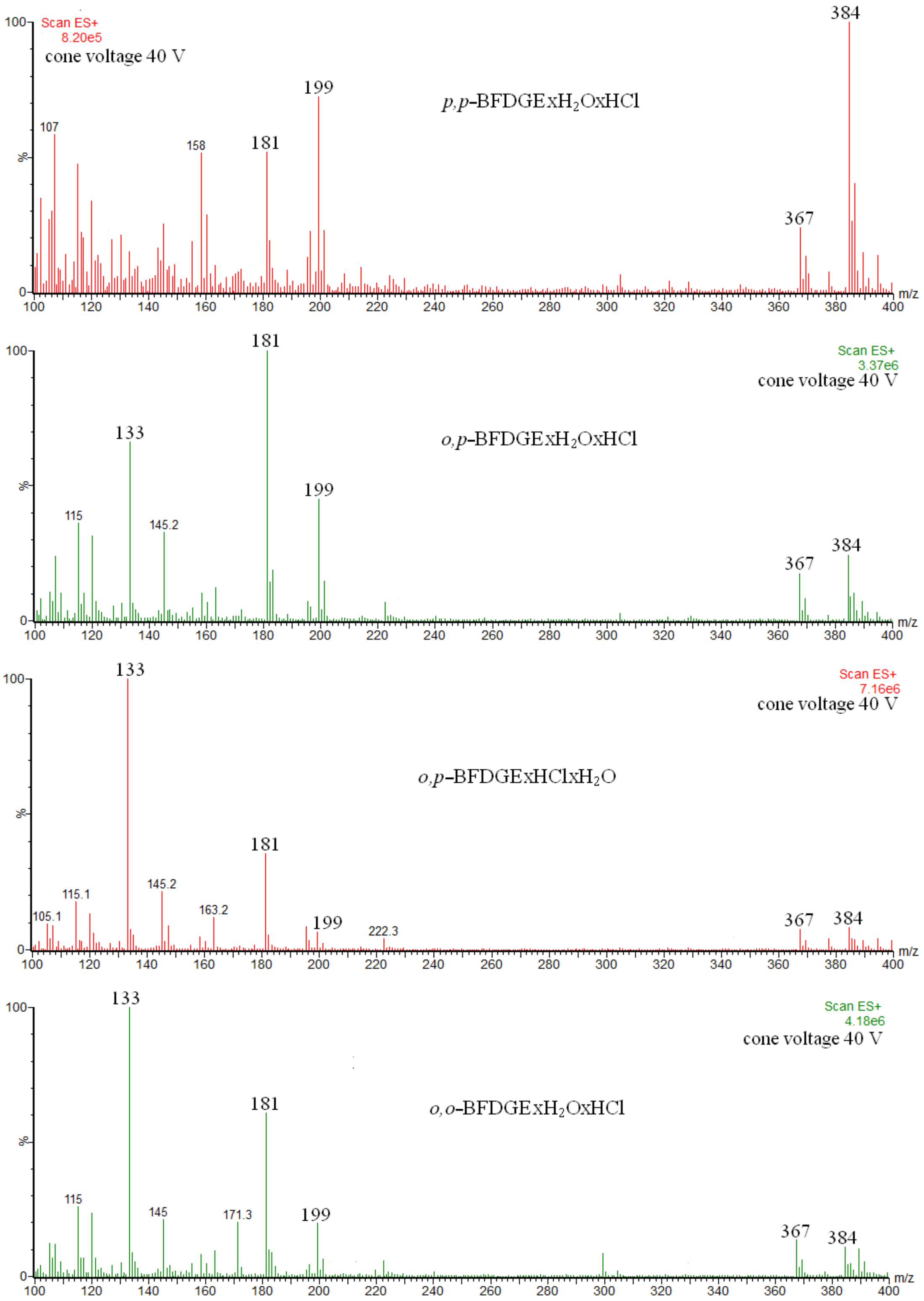

Fig. S4 ESI mass spectra of BFGDExH_2OxHCl isomers ($[M+NH_4]^+$ *m/z* 384)

GC-MS analysis

Varian CP3800 gas chromatograph with 4000MS detector - ion trap mass spectrometer (temperature: trap 180ºC, manifold 70ºC, transfer line 220ºC) and Type 1177 autosampler injector were used to carry out GC-MS analysis. The chromatographic conditions were as follows: VF-5ms 30 m x 0.25 mm i.d., 0.39 μm film thickness (Varian Part No.CP8944) column was used; the injector temperature was 220 ºC, split 1:100, and the injection volume was 1.0 μL. Helium was used as carrier gas at a constant flow rate of 1 mL/min. The oven temperature was initially set at 80 for 3 min, then increased at a rate of 15 ºC/min until 280 ºC and held at 280 ºC for 9 min. The mass spectra were obtained in full scan mode over m/z range of 40–500, under EI mode at a voltage of 70 eV.

The high stability of fragment ion at *m/z* 181 for *o,o*-BFDGE (Figure S6) can be justified by the number of resonance structures, some of them are shown in Figure S7. There is a number of examples that the "ortho-effect" involves skeletal rearrangement, namely the loss of ortho moieties is followed by cyclization [1-5]. It is clear that fragment ion at m/z 181 (shown in Figure 2) is also formed through the loss of ortho moieties accompanied by H-abstraction, followed by cyclization. For the *o,p*-BFDGE isomer the "*ortho*-effect" is manifested by the formation of the abundant fragment ion at *m/z* 197. The high stability of the fragment ion at m/z 197 for *o,p*-BFDGE can be justified by the number of resonance structures and by tautomerization, as shown in Figure S8.

References

1. D. Błachut, W. Danikiewicz, M. Olejnik, Z. Czarnocki, Electron ionization mass spectrometry as a tool for the investigation of the *ortho* effect in fragmentation of some Schiff bases derived from amphetamine analogs, J. Mass Spectrom. 39 (2004) 966-972. https://doi.org/10.1002/jms.633
2. M.A. Mendes, R.R. Rittner, M.N. Eberlin, J. Suwinski, W. Szczepankiewicz, *Ortho* effects in the dissociation of ionized N-chlorophenyl- and N-bromophenyl-2-aminobenzamidines: intramolecular aromatic substitution with cyclization to protonated 2-(2-aminophenyl)-1H-benzimidazoles, Eur. J. Mass Spectrom. 8 (2002) 27-33. https://doi.org/10.1255/ejms.470
3. D.V. Ramana, P. Mahalakshmi, Novel proximity effects and *ortho* interactions in 2,2'-disubstituted diphenylamines on electron impact, Org. Mass Spectrom. 28 (1993) 107-112. https://doi.org/10.1002/oms.1210280209

4. A.S. Płaziak, J. Spychala, H. Wojtowicz, J.J. Langer, H. Thiel-Pawlicka, K. Golankiewicz, *Ortho* effect in the mass spectrometric behaviour of N-(pyrimidin-4-yl)aminobenzoic acids and their methyl esters, Org. Mass Spectrom. 27 (1992) 1293-1298. https://doi.org/10.1002/oms.1210271124
5. F.B. Jariwala, M. Figus, A.B. Attygalle, Ortho Effect in electron ionization mass spectrometry of N-acylanilines bearing a proximal halo substituent, J. Am. Soc. Mass Spectrom. 19 (2008) 1114-1118. https://doi.org/10.1016/j.jasms.2008.05.004

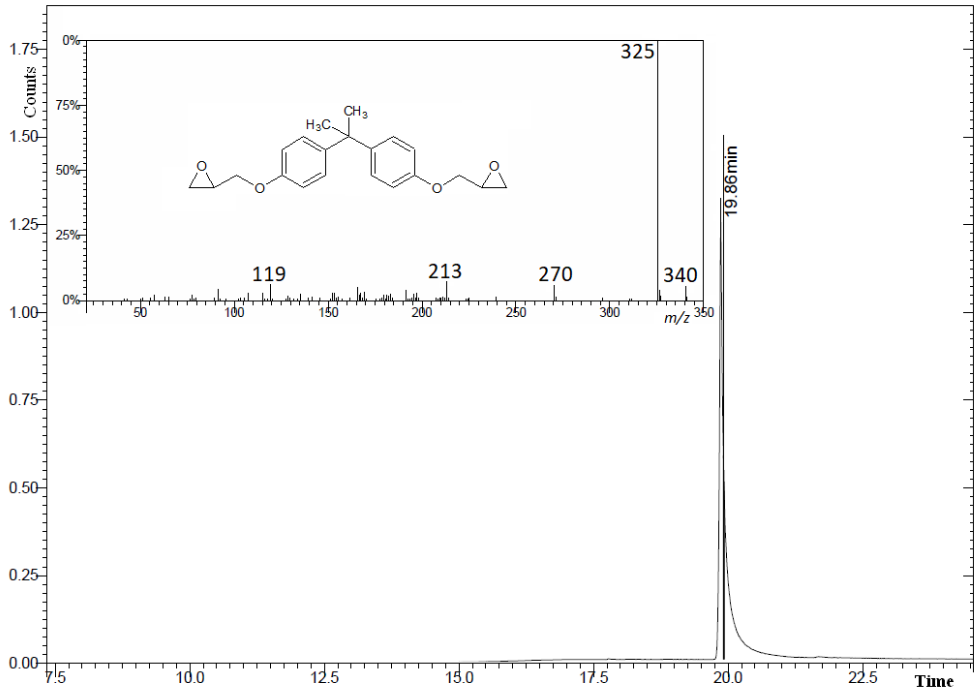

Fig. S5 Total ion chromatogram obtained for solution of BADGE and EI mass spectrum

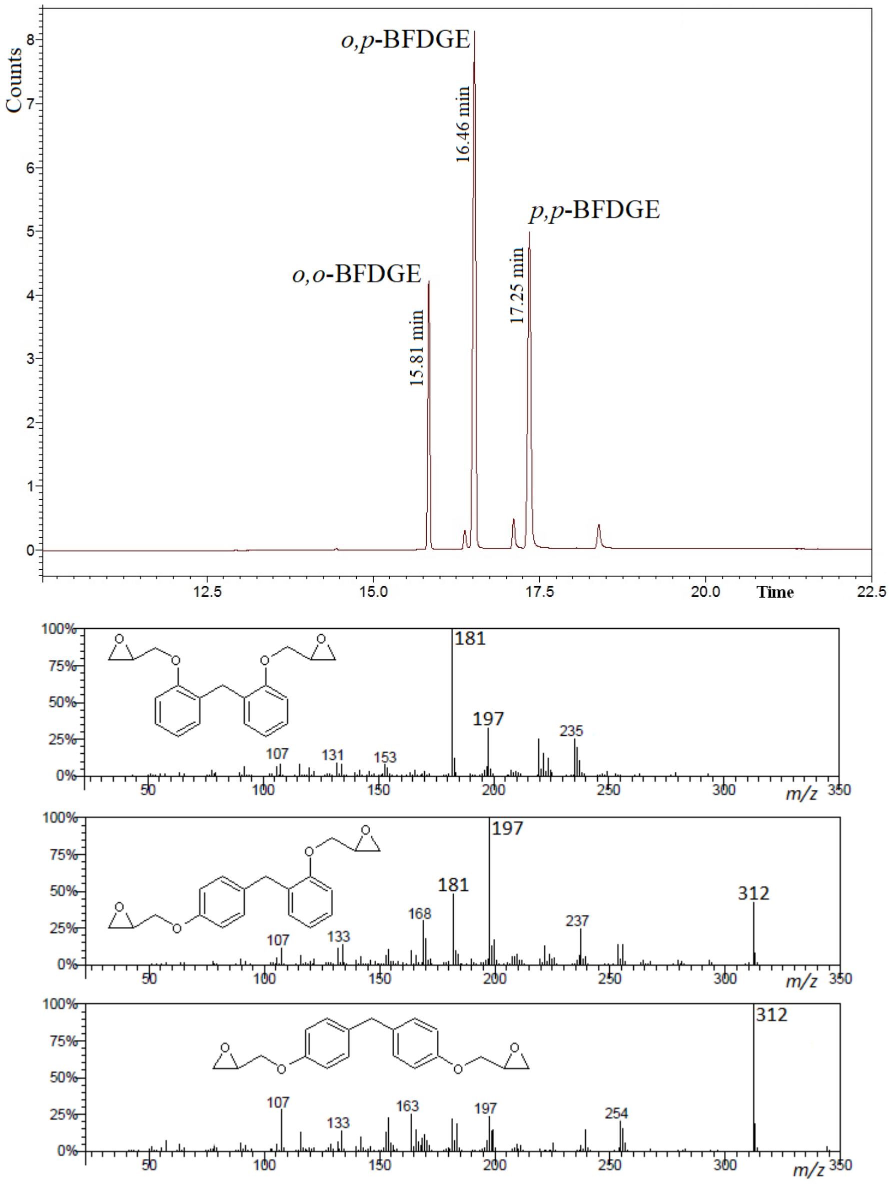

Fig. S6 Total ion chromatogram obtained for solution of BFDGE isomers and EI mass spectra obtained

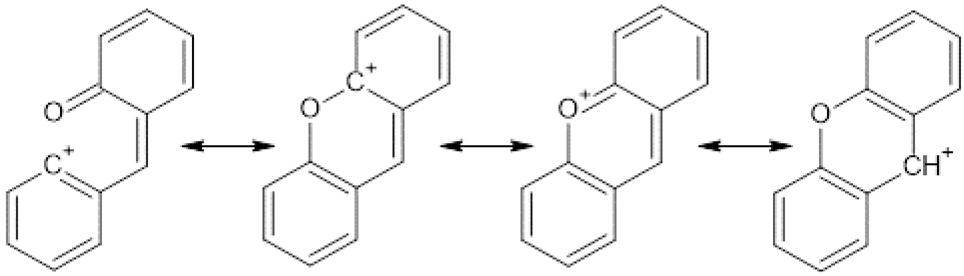

Fig. S7 Exemplary, plausible resonance structures of fragment ion at *m/z* 181 for *o,o*-BFDGE isomer

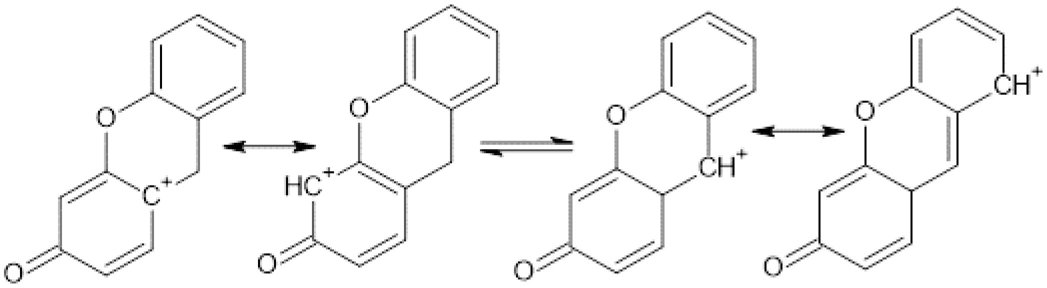

Fig. S8 Exemplary, plausible resonance/tautomeric structures of fragment ion at *m/z* 197 for *o,p*-BFDGE isomer
